# Associations among Elementary School Children’s Actual Motor Competence, Perceived Motor Competence, Physical Activity and BMI: A Cross-Sectional Study

**DOI:** 10.1371/journal.pone.0164600

**Published:** 2016-10-13

**Authors:** An De Meester, David Stodden, Ali Brian, Larissa True, Greet Cardon, Isabel Tallir, Leen Haerens

**Affiliations:** 1 Department of Movement and Sports Sciences, Ghent University, Ghent, Belgium; 2 Department of Physical Education & Athletic Training, University of South Carolina, Columbia, South Carolina, United States of America; 3 Kinesiology Department, State University of New York at Cortland, Cortland, New York, United States of America; Universita degli Studi di Catania, ITALY

## Abstract

**Background:**

Positive associations between motor competence and physical activity have been identified by means of variable-centered analyses. To expand the understanding of these associations, this study used a person-centered approach to investigate whether different combinations (i.e., profiles) of actual and perceived motor competence exist (aim 1); and to examine differences in physical activity levels (aim 2) and weight status (aim 3) among children with different motor competence-based profiles.

**Materials and Methods:**

Children’s (*N* = 361; 180 boys = 50%; *M*_age_ = 9.50±1.24yrs) actual motor competence was measured with the Test of Gross Motor Development-2 and their perceived motor competence via the Self Perception Profile for Children. We assessed physical activity via accelerometers; height through stadiometers, and weight through scales. Cluster analyses (aim 1) and MANCOVAs (aim 2 & 3) were used to analyze the data.

**Results:**

The analysis generated two predictable groups: one group displaying relatively high levels of both actual (*M* TGMD-2 percentile = 42.54, *SD* = 2.33) and perceived motor competence (*M* = 3.42, *SD* = .37; high-high), and one group with relatively low levels of both (*M* percentile = 9.71, *SD* = 3.21; *M* PMC = 2.52, *SD* = .35; low-low). One additional group was also identified as having relatively low levels of actual motor competence (*M* percentile = 4.22, *SD* = 2.85) but relatively high levels of perceived motor competence (*M* = 3.52, *SD* = .30; low-high). The high-high group demonstrated higher daily physical activity (*M* = 48.39±2.03) and lower BMI (*M* = 18.13±.43) than the low-low group (*M*_MVPA_ = 37.93±2.01; *M*_BMI_ = 20.22±.42). The low-high group had similar physical activity-levels as the low-low group (*M* = 36.21±2.18) and did not significantly differ in BMI (*M* = 19.49±.46) from the other two groups.

**Conclusions:**

A combination of high actual and perceived motor competence is related to higher physical activity and lower weight status. It is thus recommended to expand health interventions in children with components that foster the development of both actual and perceived motor competence. Health professionals should furthermore pay sufficient attention to endorsing children’s actual and perceived motor competence.

## Introduction

Regular physical activity (PA) is positively associated with physical, psychological, and social health in the short [1,2] and long-term [3,4,5]. Promoting PA also is an effective strategy to fight overweight and obesity in children and adolescents [6]. Unfortunately, children’s and adolescents’ PA levels worldwide have decreased over the last decade [7], with an alarming number of children and adolescents (75% of 11-year olds, 80% of 13-year olds and 84% of 15-year olds) not meeting the daily recommendations of at least 60 minutes of moderate to vigorous PA [8] and an increasing number of children and adolescents being overweight or obese [9]. Several potential underlying mechanisms driving PA-related behaviors and the prevention of overweight and obesity have been suggested with motor competence being identified as foundational for long-term PA engagement [10–14] and for the protection from obesity and overweight [15–17]. Motor competence is the degree of skilled performance in a wide range of motor tasks as well as the movement quality, coordination, and control underlying a particular motor outcome [18,19].

Stodden and colleagues [20] developed a conceptual model presenting relationships among motor competence, PA and the risk for obesity. The model hypothesizes motor competence and PA synergistically influence children’s weight status either positively or negatively. In the model, a positive spiral of engagement is described explaining how higher levels of motor competence and PA are related to a healthy weight status and a lower risk of obesity, whereas a negative spiral of disengagement stipulates how lower levels of motor competence and PA are associated with an unhealthy weight status and a higher risk of obesity. The relationship between motor competence and PA is suggested to strengthen with increasing age in part because of the strong mediating influence of individuals’ perceptions of their motor competence [21], which becomes more accurate as children grow older. In early childhood (2–5 years), it is expected that children’s perceived motor competence will not strongly correlate with their actual motor competence or PA since young children often lack the cognitive capability to accurately estimate their actual motor competence [22,23]. In essence, young children generally overestimate their competence levels [24]. However, as children’s cognitive capabilities continue to develop in middle (6–8 years) and late childhood (9–12 years), they become more accurate in assessing their own motor competence via more accurate comparisons against peer capabilities and their success level (or lack thereof); thus, resulting in stronger correlations between actual and perceived motor competence [24]. Indeed, during middle and late childhood stronger correlations between actual and perceived motor competence have been found in various samples [25,26,27,28] that are similar in age to the sample of the current study (7–12 years).

Until now, most studies examining the relationship between actual and perceived motor competence have used a variable-centered approach that describes and provides information on the strength of the associations between the variables [29]. A person-centered approach, on the other hand, enables the identification of groups of individuals who share particular attributes or relations among attributes [29]. These groupings may provide additional insight into children’s alignments of their actual and perceived motor competence as well as their collective association to other variables. Specifically, person-centered approaches by means of cluster analyses allow investigation of whether actual and perceived motor competence are aligned in most children or not. In other words, do most children have corresponding levels of actual and perceived motor competence (i.e., low-low and high-high) or do some children have divergent levels of actual and perceived motor competence (i.e., high-low and low-high). Studies adopting person-centered approaches to study the association between actual and perceived motor competence are scarce and are mainly conducted among adolescents. The results are furthermore incoherent to draw clear conclusions. Two studies that used a person-centered approach in a sample of American 8- to 14-year olds [30] and Belgian 12- to 15-year olds [31] identified groups with corresponding high or low levels of actual and perceived motor competence, and a group characterized by relatively lower levels of actual motor competence compared to perceived motor competence. A group with relatively higher levels of actual motor competence compared to their perceived motor competence was only identified in the Belgian study (among adolescents) [31] but not in the American study (among children in late childhood and adolescents) [30]. A person-centered approach in a sample of children in middle and late childhood could provide more clarity about the existence of a group of children in this age category with relatively higher levels of actual compared to perceived motor competence and potentially provide more insight into the findings from previous studies that used variable-centered analyses in this age group [25,32]. Therefore, the first aim of the present study was to use person-centered analyses to identify groups of children with corresponding levels (i.e., low-low or high-high) and/or divergent levels (i.e., high-low or low-high) of actual and perceived motor competence.

Person-centered analyses also may provide a more refined understanding of children’s engagement in PA and their weight status because it allows for investigation of the combined importance of actual and perceived motor competence with respect to PA and weight status. Person-centered analyses thus enable investigating whether children with different profiles of actual and perceived motor competence vary in their PA levels and weight status. While most variable-centered studies found that children with higher actual motor competence are more physically active than their less competent peers [33,34], Welk [35] suggested that children’s perceived motor competence might be even more influential than their actual motor competence. Several studies among children and adolescents indeed revealed that perceived motor competence mediates the relationship between actual motor competence and PA [21,25] and a longitudinal study among Scottish adolescents (11–15 years) found that high perceived motor competence in the final year of primary school significantly increased adolescents’ odds (by 2.5–3.8 times) of being active in the second year (girls) and the fourth year (boys) of secondary school [36]. The importance of perceived competence was confirmed in two studies that used a person-centered approach as young adolescents with high levels of perceived motor competence were more active than their peers with lower levels of perceived motor competence, even if their actual motor competence was lower [31,37]. Yet, both studies [31,37] used self-reported PA levels which increases the risk of overestimation and/or mixed method variance [38]. Therefore, the second aim of the present study was to investigate whether various motor competence-based profiles differentially predict objectively-measured PA levels in a sample of children in middle and late childhood. Thirdly, several cross-sectional [39] and longitudinal [16] studies among children and adolescents found inverse associations between actual motor competence and weight status while studies among Australian and Chinese children in late childhood and early adolescence found that obese children had significantly lower perceived motor competence than their normal weight peers [40–42]. The combined impact of children’s actual and perceived impact has, to our knowledge, not yet been studied. Therefore the third aim was to examine whether various motor competence-based profiles differentially predict children’s weight status.

In summary, the first aim of the current study was to apply person-centered analyses to examine whether different profiles based on actual and perceived motor competence could be identified in children in middle and late childhood. Based on the developmental model [20] and studies among children in late childhood and adolescents [30,31], it was hypothesized that among children of middle and late childhood at least three profiles could be identified with the majority of children having corresponding levels of actual and perceived motor competence (high-high, low-low), and a group of children having relatively lower levels of actual motor competence compared to perceived motor competence. As a fourth profile characterized by relatively higher levels of actual motor competence compared to perceived motor competence was only identified among adolescents [31] but not among children in late childhood [30], we did not assume to find such a profile in our sample of children who were in middle and late childhood.

We also aimed to investigate how children with various motor competence-based profiles differ from each other in their PA levels (aim 2) and their weight status (aim 3). We hypothesized that children with low levels of both actual and perceived motor competence would display the lowest levels of PA and have the highest risk of obesity. In contrast, children with high levels of actual and perceived motor competence were expected to have the highest levels of PA and the lowest risk of obesity [16,31,37,39].

## Materials and Methods

### Participants and procedure

Three convenience samples (*N* = 361; 180 boys; 49.86%) with a mean age of 9.49 years (*SD* = 1.24, range 6.92–11.83 years) were included in this cross-sectional study. One sample (*n* = 64; 59% boys) was selected from an urban school district in Ohio and was predominantly non-Hispanic White. The second sample (*n* = 196; 54% boys) was selected from a rural school in Texas and was 58% Hispanic with the remaining children being predominately non-Hispanic White. The third sample (*n* = 101; 55% boys) was recruited from a before and after school program in Michigan and consisted of predominantly non-Hispanic White children. Prior to participation, permission was obtained from the school districts and Institutional Review Boards from each of the three geographical locations (the Ohio State University Institutional Review Board, the Michigan State University Institutional Review Board, and the Texas Tech University Institutional Review board) to conduct the study. After written parental consent was obtained, a member of the research team read the assent form for the children out loud in small groups. Children were then asked to circle a smiley face and write their name if they wanted to participate or to circle a frowny face if they did not want to participate. Two children wished not to participate in the present study. They were given an alternate activity during the Physical Education class in which data from the children who gave assent was collected.

Youth with any physical disability or health condition that prevented completion of any of the assessments were not allowed to participate in testing.

### Measures

#### Actual motor competence

Actual motor competence was measured using the Test of Gross Motor Development—second edition (TGMD-2) [43]. The TGMD-2 is a valid and reliable norm-referenced measure assessing six locomotor skills (run, gallop, hop, leap, horizontal jump, slide) and six object-control skills (striking a stationary ball, stationary dribble, kick, catch, overhand throw, and underhand roll). The TGMD-2 was administered at school by a team of researchers who followed the standard testing procedure which took approximately 15 minutes per child. In line with the procedures, children were given two trials per skill and each skill had between 3 to 5 components that needed to be demonstrated for the skill to be performed proficiently. The scores of both trials were summed to obtain a raw score for each skill. The six locomotor skill scores and the six object-control skills were summed to provide an overall score which was then converted to a percentile score, standardized for age and sex [43].

#### Perceived motor competence

Similar to previous studies [21,30], the sport/athletic competence subscale (i.e., one’s ability to do well at sports) of the Self-Perception Profile for Children (SPCC) [44] was used to assess children’s perceptions of their athletic ability and their ability to learn sports skills. The SPPC is a highly reliable (internal consistency reliability of 0.71 ≤ r ≤ 0.91 for the different subscales) and valid instrument to assess different dimensions of self-perception among children [45]. The SPPC was conducted one-on-one in a semi-private setting (i.e., in a hallway or an empty classroom) to protect the privacy of the child and to avoid reporter bias. A member of the research team asked each child if he/she wanted to read the items himself/herself or if he/she preferred for the member of the research team to read it to him/her. The administration of the SPPC took approximately five minutes per child. Answering categories of the sport/athletic competence subscale (6 items) consist of a four-choice structured alternating format to minimize socially desirable responses [46]. The child is first asked to decide with which kind of child he or she identifies most, the one(s) described in the first part of the sentence or the one(s) described in the second part of the sentence (e.g., ‘Some children do very well at all kinds of sports but other children don’t feel that they are very good when it comes to sports.’). Once having made this decision, the child then decides whether the description in the part of the sentence he/she chose is “really true” or “sort of true” for him/her. Each item was accordingly scored from 1 (low perceived competence) to 4 (high perceived competence). The scores of the 6 items were summed and then divided by 6, providing a minimum score of 1 and a maximum score of 4 [44].

#### Physical activity

Five-day daily PA-levels were assessed using ActiGraph GT3X+ accelerometers (Manufacturing Technologies Inc., Shalimar, FL). Children were instructed to wear the accelerometer on the right hip during waking hours and to remove it only for sleeping and water-based activities (e.g., showering or swimming). Children wore accelerometers for a minimum of five days (3 week days and 2 weekend days) and all the accelerometers collected data over 15 seconds epoch time intervals. Cutoff points for physical activity in youth (6–16 years) as defined by Evenson et al. [47] were used. The sum of moderate (2296–4011 counts) and vigorous (>4012 counts) PA (MVPA) was used in the present study. Acceptable inclusion criterion for wear time was at least nine hours per day [48]. Compliance with wearing accelerometers was facilitated by guidelines previously published [49].

#### Anthropometry

Standing height was measured to the nearest 0.1 cm using portable stadiometers (Shorr Productions; Olney, MD). Mass was measured to the nearest 0.1 kg using portable scales (Seca, Model 770, Hamburg, Germany). Height and mass measurements were taken twice and the average of the two measurements was retained for analysis. If height or mass measurements differed by more than 0.5 cm or 0.5 kg, respectively, a third measurement was taken and the two closest measurements were averaged. Body Mass Index (BMI) was calculated to determine children’s weight status by dividing body mass (in kg) by height squared (in m) [50] while controlling for sex and age.

### Analyses

**Fig 1:**
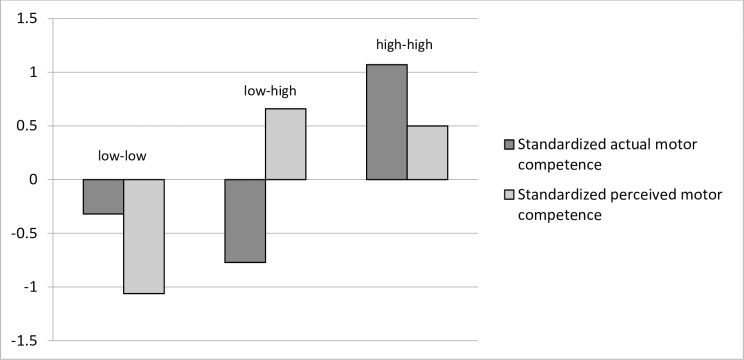
Three cluster solution based on z-scores for actual motor competence and perceived motor competence.

All statistical analyses were conducted in IBM SPSS Statistics 22.0. Statistical significance was set at *p* < .05. To examine whether different profiles based on actual and perceived motor competence could be identified cluster analyses were conducted based on standardized scores of children’s actual and perceived motor competence. After removing one univariate outlier (which had a value of more than three standard deviations below the mean) and one multivariate outlier (as identified using the Mahalanobis distance measure) [51], a two-step procedure using a combination of hierarchical and non-hierarchical clustering methods [52] was applied. Ward’s hierarchical clustering method was conducted [53] to combine clusters that were similar in terms of squared Euclidean distance. This resulted in three-, four, and five-cluster solutions. As the explained variance in both motor competence dimensions of each cluster solution was at least 50%, all the cluster solutions were retained for the following step [54] in which the cluster centers were used as non-random initial cluster centers in an iterative, non-hierarchical k-means clustering procedure [55]. To examine the stability of the cluster solutions, a double-split cross-validation procedure was implemented [56] by randomly splitting the total sample into halves and applying the two-step procedure (Ward and k-means) in each subsample. The participants in each half of the sample were then assigned to new clusters based on their Euclidean distances to the cluster centers of the other half of the sample. These new clusters were then compared for agreement with the original clusters by means of Cohen’s kappa (K). The two resulting kappas were averaged and a Cohen’s kappa of at least .60 (good agreement) was considered acceptable [55]. Stability and replicability was acceptable only for the three-cluster solution with a kappa of .79. The four- and five-cluster solution had a kappa of .50 and .39, respectively. The four- and the five- cluster solution explained more variance in actual and perceived motor competence than the three-cluster solution. Since the difference in explained variance was only small (less than 15%) and stability of the four- and five-cluster solution was low, it was decided to only retain the three-cluster solution for further interpretation. Fig 1 represents the final three-cluster solution, which accounted for 58% of the variance in actual motor competence and 54% of the variance in perceived motor competence. To define each of the clusters, standardized mean scores of both actual and perceived motor competence were inspected. We also looked at the absolute mean scores to situate the clusters with respect to normative data.

To investigate differences in MVPA and BMI among children with various motor competence-based profiles a MANCOVA was conducted. Since sex was significantly related to MVPA and age to BMI, we controlled for both variables. The least square means procedure was used to detect significant subgroup differences.

## Results

### Descriptives

The means and standard deviations of the variables, as well as the correlation coefficients among these variables, are presented in Table 1. Children had low overall levels of actual motor competence with a mean percentile score of 18.97 (*SD* = 21.78) and they reported high levels of perceived motor competence with an average of 3.14 (*SD* = .57) on a four-point scale. Boys (*M*_*percentile*_ = 18.24, *SD* = 20.66) did not significantly differ from girls (*M*_*percentile*_ = 19.69, *SD* = 22.89) with respect to actual motor competence (*F*[1] = 0.17, *p* = .69) but there was a significant yet small difference (*F*[1] = 4.25, *p* = .04) between boys (*M* = 3.20, *SD* = .53) and girls (*M* = 3.09, *SD* = .61) in terms of perceived motor competence. Boys were found to be significantly more physically active than girls (*F*[1] = 35.70, *p* < .001) with boys engaging on average 48.17 minutes per day in MVPA (*SD* = 25.91) and girls averaging 33.95 minutes per day (*SD* = 16.27). In total, 16.21% of the children (23.27% of the boys and 9.52% of the girls) met the guidelines of 60 minutes or more MVPA per day. Children had an average BMI of 19.34 (*SD* = 4.65). Boys’ BMI did not significantly differ from girls’ (*F*[1] = 0.35, *p* = .56). Almost one third of the children (29.44%) was overweight or obese.

**Table 1:** Descriptive statistics and correlations among variables.

*Variables*	*M*	*SD*	*Min*	*Max*	*1*	*2*	*3*	*4*
1. Actual motor competence (percentile)	18.97	21.78	1.00	92.00				
2. Perceived motor competence (1–4 scale)	3.14	.57	1.50	4.00	.20***			
3. Moderate to vigorous physical activity (min/day)	40.86	22.62	5.00	160.00	.31***	.17**		
4. Body Mass Index (kg/m^2^)	19.34	4.65	11.90	41.97	-.11*	-.19***	-.16**	
5. Age (years)	9.50	1.24	6.92	11.83	.29***	-.33***	.04	.26***

*Note*. N = 361 children (180 boys) (except for MVPA where n = 327; 159 boys).

*p < .05

**p < .01

***p < .001.

### Identifying profiles based on actual and perceived motor competence

Three different motor competence-based clusters, all approximately the same size, were identified in the present sample (Table 2; Fig 1). The clusters were labelled based on relative scores for actual motor competence (high vs. low) and perceived motor competence (high vs. low), respectively. Children in cluster 1 (*n* = 123; 34.26%) had, relative to children belonging to the other clusters, low levels of both actual and perceived motor competence. This cluster was labeled the ‘low-low’ cluster. Cluster 2 (*n* = 115; 32.03%) was characterized by children who had, relative to children belonging to the other clusters, low levels of actual motor competence but high levels of perceived motor competence and was labeled the ‘low-high’ cluster. Children in cluster 3 (*n* = 121; 33.70%) had, relative to children belonging to the other clusters, high levels of both actual and perceived motor competence. This cluster was labeled the ‘high-high’ cluster. Boys and girls were equally distributed across clusters (χ^2^ = 7.87; *df* = 1; *p* = 0.89) with 43.09% boys in the low-low cluster (*n* = 53), 57.40% boys in the low-high cluster *n* = 66), and 49.59% boys in the high-high cluster (*n* = 60). Significant differences in actual motor competence were found among the three clusters (Table 1). The low-high cluster had the lowest mean score for actual motor competence (*M*_*percentil e*_ = 4.22, *SD* = 2.85), followed by the low-low cluster (*M*_*percentile*_ = 9.71, *SD* = 3.21) and the high-high cluster (*M*_*percentile*_ = 42.54, *SD* = 2.93) respectively. Even though children in the high-high cluster had relatively high levels of actual motor competence when compared to the other children in the sample, in absolute terms (i.e., when compared to the normative data for their age as defined by Ulrich) [43], they scored rather average with a mean percentile score of 42.54. Children in the other two clusters had a low actual motor competence both in relative and absolute terms (Table 1). There were also significant differences in perceived motor competence with the low-low cluster (*M* = 2.52, *SD* = 0.35) having a lower mean than the low-high (*M* = 3.52, *SD* = 0.30) and the high-high cluster (*M* = 3.42, *SD* = 0.37). Children in the relatively low-low cluster had an average perceived motor competence coinciding with the 2.5 midpoint of the four-point scale, suggesting that these children (despite having low levels of perceived motor competence in comparison with the other children in the study sample) perceive themselves as average.

**Table 2:** Mean scores and cluster comparisons for the three Clusters (N = 359): actual and perceived motor competence.

Variable	Cluster				
	Cluster 1: Relatively low—low	Cluster 2: Relatively low—high	Cluster 3: Relatively high—high	F	η^2^
	n = 123 53 boys, 70 girls 34.26%	n = 115 66 boys, 49 girls 32.03%	n = 121 60 boys, 61 girls 33.70%		
**Cluster dimensions (z-scores)**					
Actual motor competence	-0.32 (0.67)^b^	-0.77 (0.59)^a^	1.07 (0.61)^c^	273.05***	0.61
Perceived motor competence	-1.06 (0.60)^a^	0.66 (0.52)^b^	0.50 (0.63)^b^	319.24***	0.64
**Cluster dimensions (raw scores)**					
Actual motor competence (percentile)	9.71 (3.21)^b^	4.22 (2.85)^a^	42.54 (2.93)^c^	273.05***	0.61
Perceived motor competence	2.52 (0.35)^a^	3.52 (0.30)^b^	3.42 (0.37)^b^	319.24***	0.64

*Note*. Values in parentheses are standard errors. A cluster mean is significantly different from another mean if they have different superscripts (i.e., a, b and c).

***p < .001.

### Differences between motor competence-based profiles in MVPA and BMI

The MANCOVA (controlled for age and sex) showed significant differences among clusters for both MVPA (*F*[2] = 10.28; *p* < 0.001) and BMI (*F*[2] = 6.44; *p* = 0.002) with children in the high-high cluster demonstrating a significantly higher MVPA (48.39, SE = 2.03) than children in the low-low cluster (37.93, SE = 2.01) and the low-high cluster (36.21, SE = 2.18). Children in the high-high cluster demonstrated a significantly lower BMI (18.13, SE = .43) than children in the low-low cluster (20.22, SE = .42). Children in the low-high cluster (19.49, SE = .46) did not significantly differ in their BMI from children of the other two groups (see Table 3).

**Table 3:** Mean scores and cluster comparisons for the three Clusters (N = 327): physical activity and BMI.

Variable	Cluster				
	Cluster 1: Relatively low—low	Cluster 2: Relatively low—high	Cluster 3: Relatively high—high	F	η^2^
Moderate to vigorous physical activity (min/day)	37.93 (2.01)^a^	36.21 (2.18)^a^	48.39 (2.03)^b^	10.27***	0.06
Body Mass Index (kg/m2)	20.22 (0.42)^a^	19.49 (0.46)^ab^	18.13 (0.43)^b^	6.44**	0.04

*Note*. Values in parentheses are standard errors. A cluster mean is significantly different from another mean if they have different superscripts (i.e., a and b). All values are controlled for age and sex.

**p < .01

***p < .001.

## Discussion

The present study used a person-centered approach to examine whether different profiles based on actual and perceived motor competence could be identified in children in middle and late childhood. It also was investigated how children with various motor competence-based profiles differ from each other in their PA levels and their weight status. Cluster analyses revealed two groups of children with corresponding levels of actual and perceived motor competence (i.e., low-low and high-high) and one group with divergent levels of actual and perceived motor competence (i.e., low-high). A group of children with relatively higher levels of actual motor competence compared to perceived motor competence was not identified. These findings are in line with a study among American 8- to 14-year olds that identified the same motor competence-based profiles [30]. A fourth profile, as identified in a recent study among Belgian 12- to 15-year old adolescents [31], characterized by relatively higher levels of actual motor competence compared to perceived motor competence, was not found in the current study. This may indicate that a combination of relatively high levels of actual but low levels of perceived motor competence is a rare phenomenon in children while it is more common in adolescents. The lack of such a profile in the current study could also partially be explained by the absolute values of the children’s actual (mean percentile score = 18.97) and perceived motor competence (mean on a 1–4 scale = 3.14). Because most children had low actual motor competence, it might become more challenging to identify a high-low group. At the same time, the overall high levels of perceived motor competence in this group of children with low actual motor competence may indicate that children in middle and late childhood, despite being able to more accurately assess their own motor competence than children in early childhood [24], still tend to overestimate their actual motor competence. In adolescents, this seems to be less the case [31]. This is in line with previous research that found that general self-esteem is higher among elementary school children than adolescents [57,58].

The results further indicate that children in the low-low group were significantly less physically active and had a significantly higher BMI than children in the high-high group. Children in the low-high group also demonstrated lower PA levels than the high-high group while their BMI did not significantly differ from children in the high-high group. No differences in BMI were found between the low-high and the low-low group. The large difference in objectively measured PA between the low-low group and the high-high group confirms the findings of previous studies that used self-reported PA [31,37] in samples of 7^th^ and 8^th^ grade students. However, it is striking that the low-high group is not more physically active than the low-low group. Based on the findings of De Meester et al. [31] and the mediating role of perceived motor competence in the relationship between actual motor competence and physical activity [21,25], it was expected that children in the low-high group would have higher PA levels than children in the low-low group. The current findings indicate that perceived motor competence (although significantly related to MVPA in the total sample) among children with a very low actual motor competence might be less critical to PA in middle and late childhood compared to adolescence [20]. The rather large difference in PA (more than ten minutes per day) between children with relatively high levels of actual motor competence (i.e., high-high) and children with low levels of actual motor competence (i.e., low-low and low-high) highlights the importance of developing children’s actual motor competence to obtain a physically active lifestyle. It furthermore offers perspectives to improve existing interventions aiming at increasing children’s PA. In groups with overall low levels of actual motor competence, it might be less effective to focus on perceived motor competence, if children’s actual motor competence is not improved first. We know from previous studies that children’s actual motor competence can be improved by means of motor skill interventions [59]. The difference in PA (i.e., 10–11 minutes/day) between children in the high-high group and the groups with low actual motor competence (i.e., low-low, low-high) is comparable to the difference in PA promoted by the most successful multicomponent PA interventions with children [60,61]. Based on these data, specifically focusing on context-specific movement activities that are developmentally appropriate and foster the development of children’s actual (and perceived) motor competence, might contribute to higher and sustainable increases in PA as a result of the interventions [62].

In addition to being more physically active, children in the high-high group were also found to have a lower BMI than children in the low-low group. These results are in line with previous studies that found negative associations between children’s BMI and their actual motor competence [16,39,63]. Less than a quarter of the children in the high-high group were obese or overweight (23.14%) while 31.30% of the children in the low-high group and 33.87% of the children in the low-low group were found to be obese or overweight. However, children in the low-high group did not significantly differ in their BMI from children in the low-low or the high-high group. These findings suggest that the cumulative effect of the combination of children’s actual and perceived motor competence (rather than solely their actual motor competence) may be more predictive of weight status. This interrelationship among children’s actual motor competence, perceived motor competence and weight status provides cross-sectional evidence for the associations between the three variables as proposed in the conceptual model [14,20]. Although it seems that, in terms of PA, perceived motor competence is a less critical factor among children with low levels of actual motor competence, it seems to play a far more important role with regard to children’s weight status.

### Strengths, limitations and future research

One limitation of the present study is its cross-sectional design. The results do not provide causal evidence regarding relationships among actual motor competence, perceived motor competence, PA, and weight status. To gain more insight in the direction of these relationships and to understand how associations among these variables may change over time, longitudinal or experimental studies should be conducted. Another limitation is the use of a convenience sample which can lead to the under-representation or over-representation of particular groups within the sample. Even though the total sample consisted of a large and diverse sample of 361 children from three different States, it should be noted though that the majority of the children in the current study (52%) had a low socio-economic status. This might partially explain the overall low actual motor competence of this sample since previous studied found a negative effect of social disadvantage on children’s actual motor competence [64,65].

The present study also had some considerable strengths with the first being the use of person-centered analyses. This innovative approach sheds new light on the findings of previous studies that mainly used a variable-centered approach. Another strength is the use of objective measurements of both BMI (stadiometers and scales) and PA (accelerometry). These objective measurements exclude the risk of overestimation and/or mixed method variance [38].

## Conclusion

The results of the present study showed that a combination of relatively high levels of actual and perceived motor competence in children is related to considerably higher daily MVPA levels and a lower BMI. Intuitively, as the overall “average” level of actual motor competence noted in the “high” group was only at the 48 percentile (based on TGMD-2 normative data), it would be interesting to see whether a group of actual “high functioning” children (e.g., 75 percentile) would demonstrate even higher PA levels and a lower BMI. It would also be interesting to investigate whether the role of perceived motor competence would be different, among children with higher levels of actual motor competence. Thus, further inquiry in this line of work is required. It is furthermore recommended to expand or adapt health-interventions in children with components that foster the development of both actual and perceived motor competence as it may significantly improve the long-term impact of the intervention. Finally, health professionals can try to pay sufficient attention to endorsing children’s actual and perceived motor competence.
